# The (Pro)renin Receptor in Kidney Development: Cellular, Molecular, and Signaling Mechanisms

**DOI:** 10.3390/cells15212024

**Published:** 2026-11-06

**Authors:** Renfang S. Taylor

**Affiliations:** Department of Natural Sciences, Anderson University, 1100 E 5th St, Anderson, IN 46012, USA; rstaylor@anderson.edu

**Keywords:** (pro)renin receptor, ATP6AP2, kidney development, nephrogenesis, ureteric bud, soluble (P)RR, Wnt/β-catenin, V-ATPase, epigenetic regulation

## Abstract

**Highlights:**

**What are the main findings?**

- The (pro)renin receptor ((P)RR/ATP6AP2) has multifaceted roles in nephrogenesis, including ureteric bud branching, nephron progenitor maintenance, epithelial–mesenchymal interactions, and collecting duct development.
- ATP6AP2 loss-of-function models demonstrate developmental requirements, whereas ligand-dependent receptor signaling, V-ATPase-associated functions, and proposed downstream pathways must be evaluated separately; GDNF/RET and Notch links remain hypotheses.

**What are the implications of the main findings?**

- Developmental ATP6AP2 perturbation can impair renal morphogenesis; any subsequent contribution to adult hypertension or chronic kidney disease remains an untested developmental-programming hypothesis.
- Soluble (P)RR, kidney organoids, single-cell approaches, and epigenetic profiling provide emerging avenues for mechanistic and translational investigation.

**Abstract:**

The (pro)renin receptor ((P)RR), encoded by ATP6AP2, participates in kidney development and renal physiology through several potentially distinct activities: ligand-associated signaling, V-ATPase-related cellular functions, and generation of soluble (P)RR (s(P)RR). Experimental lineage-targeted ATP6AP2 perturbation supports requirements in ureteric bud branching and stromal–epithelial interactions, and a ureteric bud deletion model implicates Dot1/H3K79-associated changes. Nevertheless, deletion phenotypes do not by themselves establish that renin/prorenin binding causes the developmental defects. Wnt/V-ATPase interactions provide a mechanistic framework, whereas proposed links to GDNF/RET, Notch, PI3K/AKT, and metabolic pathways vary in evidentiary support and should not be interpreted as proven kidney-specific regulatory axes. Evidence for s(P)RR in sodium handling and adult renal injury must likewise be distinguished from its uncertain role in nephrogenesis. This review evaluates the strength and limitations of experimental findings, compares available developmental models, and outlines approaches to distinguish ligand-dependent actions from ATP6AP2/V-ATPase-associated functions. An association between abnormal nephrogenesis and later kidney disease is biologically plausible, but direct longitudinal evidence following developmental ATP6AP2 disruption remains limited.

## 1. Introduction

Kidney development is a highly coordinated process involving reciprocal interactions among the ureteric bud, metanephric mesenchyme, stromal progenitors, and nephron progenitor cells [1,2,3,4]. Nephrogenesis requires precise regulation of signaling pathways controlling branching morphogenesis, nephron induction, cellular differentiation, and organ maturation [1,2,5]. Disruption of these developmental processes may result in congenital anomalies of the kidney and urinary tract (CAKUT), reduced nephron endowment, and increased susceptibility to hypertension and chronic kidney disease later in life [2,6].

The renin–angiotensin system (RAS) plays a critical role during kidney development, particularly in ureteric bud branching and nephron formation [1,2]. Among RAS-associated proteins, the (pro)renin receptor ((P)RR), encoded by ATP6AP2, has emerged as an important developmental regulator with functions extending beyond classical renin signaling [7,8,9,10]. Initially identified as a receptor for renin and prorenin, (P)RR has since been shown to participate in intracellular signaling, vacuolar H+-ATPase (V-ATPase) regulation, Wnt/β-catenin signaling, and developmental gene regulation [7,11,12,13].

Recent studies have demonstrated that (P)RR is critically involved in kidney organogenesis, ureteric bud branching morphogenesis, stromal signaling, and nephron progenitor maintenance [1,2,14,15,16]. In addition, soluble (P)RR (s(P)RR) has emerged as a potential regulator of renal sodium handling, fibrosis, hypertension, and kidney injury [17,18,19,20]. Advances in kidney organoids, epigenetic profiling, and single-cell sequencing technologies have further expanded understanding of the cellular and molecular mechanisms underlying (P)RR function during nephrogenesis [21,22,23,24,25,26].

This review evaluates (P)RR/ATP6AP2 biology by distinguishing (i) experimentally observed phenotypes following ATP6AP2 perturbation, (ii) biochemical or cellular associations, and (iii) proposed kidney-specific mechanisms. These distinctions are examined throughout the manuscript, with particular attention to the strength and limitations of the available evidence. The term “direct” is reserved for a mechanistic relationship tested in an appropriate model, rather than merely the occurrence of a phenotype following gene deletion.

## 2. Structure and Biology of the (Pro)renin Receptor

### 2.1. Discovery and Structure of (P)RR

**Figure 1:**
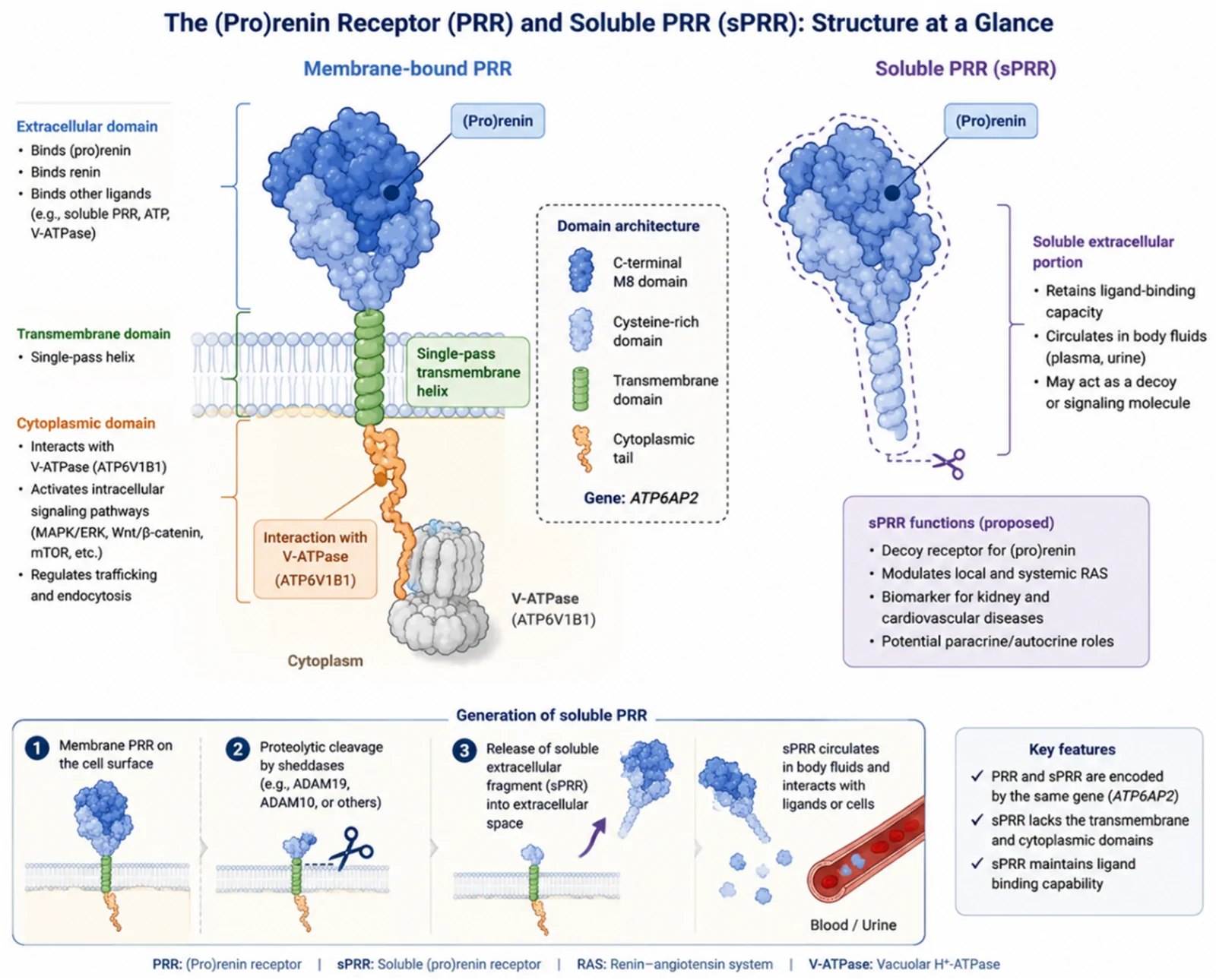
Schematic illustration of the membrane-bound (pro)renin receptor ((P)RR) and soluble (P)RR (s(P)RR). Membrane-bound (P)RR contains extracellular, transmembrane, and intracellular domains and is associated with vacuolar H+-ATPase (V-ATPase). Proteolytic cleavage of membrane-bound (P)RR by specific proteases generates soluble (P)RR, which is subsequently released into extracellular fluids and circulation. The figure highlights the structural organization of (P)RR and the process involved in s(P)RR generation. AI-assisted figure generation: ChatGPT-4 (OpenAI, San Francisco, CA, USA); scientific content and accuracy reviewed by the author.

The structural organization of membrane-bound (P)RR and the generation of soluble (P)RR are summarized in Figure 1. The (pro)renin receptor was first identified as a receptor capable of binding both renin and prorenin, thereby activating intracellular signaling pathways independently of angiotensin II generation. Previous studies demonstrated that binding of prorenin to (P)RR induces conformational activation of prorenin and stimulates intracellular signaling through mitogen-activated protein kinase (MAPK) pathways [7,8,9]. Subsequent studies identified (P)RR as ATP6AP2, a multifunctional protein associated with vacuolar H+-ATPase complexes and intracellular trafficking.

(P)RR exists in both membrane-bound and soluble forms. Membrane-bound (P)RR participates in intracellular signaling and developmental regulation, whereas soluble (P)RR is generated through proteolytic cleavage and released into extracellular fluids. Soluble (P)RR has increasingly been recognized as a biologically active molecule involved in renal sodium handling, fibrosis, and hypertension.

The receptor contains extracellular, transmembrane, and intracellular domains that facilitate interactions with renin, prorenin, V-ATPase components, and intracellular signaling complexes. Multiple studies highlighted the diverse interaction partners and multifunctional cellular roles of (P)RR beyond traditional RAS activation [11,12,13].

Mechanistic distinction: ATP6AP2 deletion removes more than a ligand-binding receptor activity. As an accessory protein associated with V-ATPase, ATP6AP2 can affect organelle acidification, membrane trafficking, protein turnover, and cell viability. Consequently, developmental defects in conditional knockout animals should not be attributed specifically to renin/prorenin-triggered signaling without ligand-binding-selective perturbations, rescue experiments, or separation-of-function alleles [11,12,13].

Three distinct claims are often grouped under the label “(P)RR signaling,” although they require different experimental demonstrations. Ligand-dependent signaling requires evidence that renin or prorenin binding changes a defined cellular response and that selective interference with binding reverses it. ATP6AP2-associated cellular function refers to effects on V-ATPase assembly or operation, vesicular pH, and membrane traffic that can occur without a demonstrated extracellular ligand requirement. Finally, soluble (P)RR is a proteolytic product with reported physiological activities, but its production and function should not be treated as interchangeable with those of full-length ATP6AP2. Gene deletion can perturb more than one category simultaneously. Thus, attributing a deletion phenotype to one activity requires appropriate controls and, ideally, a validated separation-of-function rescue. This distinction is especially consequential in the developing kidney, where epithelial polarization, extracellular signaling, and cell survival are tightly interdependent.

Megalin-mediated renin/prorenin uptake has also been reported, emphasizing that ligand internalization and trafficking should not automatically be attributed to ATP6AP2-dependent signaling [27].

### 2.2. (P)RR and Intracellular Signaling

Although initially linked to renin signaling, (P)RR is now recognized as an important mediator of intracellular signaling pathways critical for development and cellular homeostasis [7,8,9,10,11,12,13]. Binding of prorenin to (P)RR activates MAPK/ERK pathways, promoting cellular proliferation, fibrosis, and inflammatory signaling [7,8,9,10,28,29,30,31,32,33].

In addition, (P)RR functions as an accessory component of the vacuolar H+-ATPase complex, which regulates intracellular acidification, vesicle trafficking, and cellular metabolism [11,12,13]. Disruption of V-ATPase-associated signaling may impair developmental regulation and nephron differentiation [11,12,13].

(P)RR also participates in Wnt/β-catenin signaling, an essential pathway during kidney development [1,2,3,4,5,6,23,24,25,26,34]. Wnt signaling regulates nephron induction, progenitor maintenance, branching morphogenesis, and epithelial differentiation. Experimental studies suggest that deletion of (P)RR disrupts developmental signaling pathways involved in nephrogenesis and collecting duct development [1,2,14,35].

### 2.3. Soluble (P)RR

Soluble (P)RR has emerged as an increasingly important area of investigation in renal physiology and pathology. Evidence from experimental studies indicates that s(P)RR can influence renal sodium transport, collecting duct signaling, fibrosis, and intrarenal RAS activation [17,18,19,20]. These findings support biological activity beyond its potential use as a biomarker, although its functions are context dependent and its role during kidney development remains uncertain.

Experimental studies demonstrated sex-specific cardiorenal effects of human soluble (P)RR expressed in mouse collecting ducts, suggesting important physiological and pathological functions of s(P)RR [36]. Additional studies identified soluble (P)RR as a novel regulator of renal medullary sodium reabsorption [20].

The biological role of s(P)RR during kidney development remains incompletely understood. Its established actions in renal physiology and disease should therefore be distinguished from proposed developmental functions. Determining whether s(P)RR directly influences nephrogenesis, developmental cell–cell communication, or later disease susceptibility will require dedicated developmental studies.

## 3. (P)RR in Kidney Development

**Figure 2:**
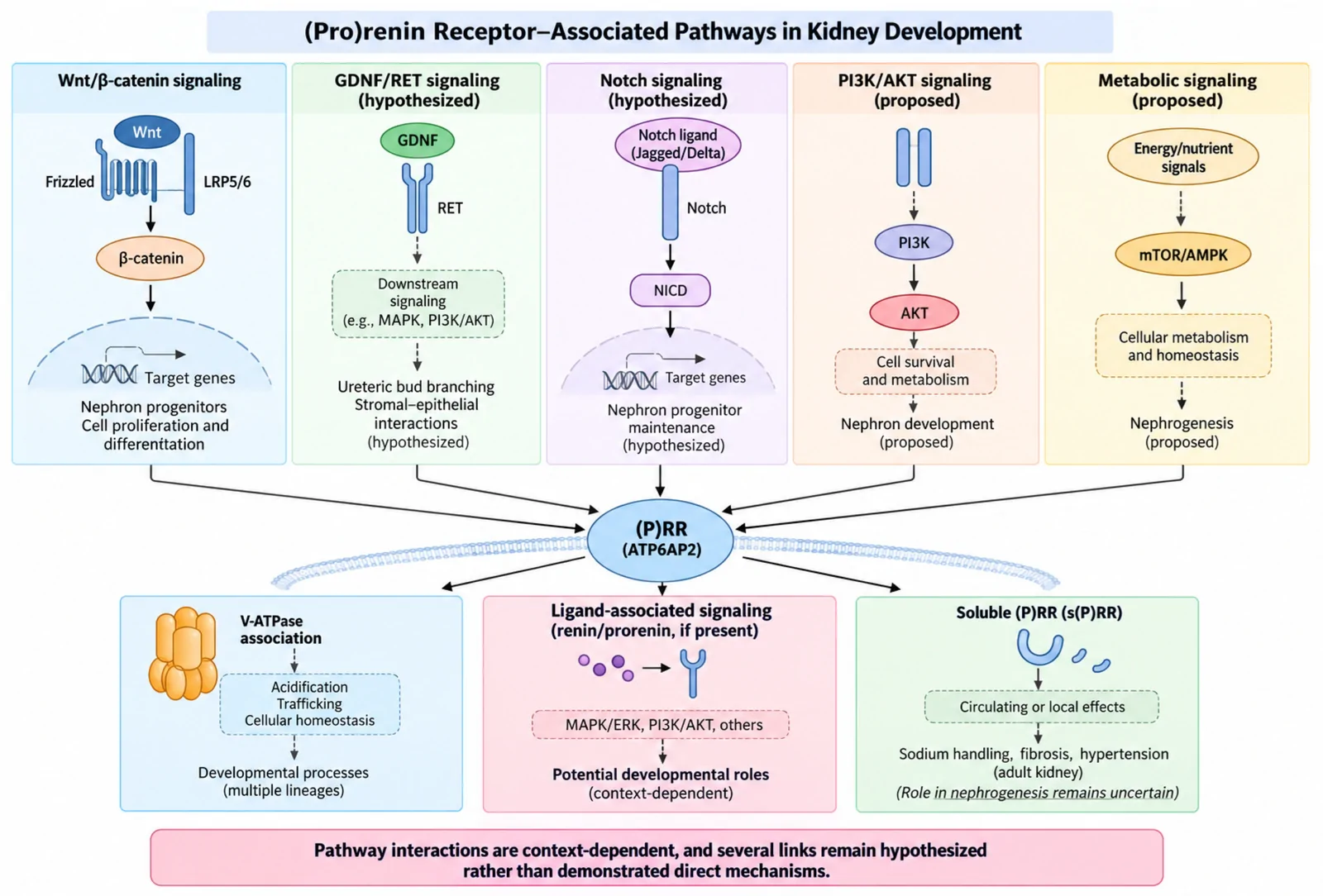
Evidence-informed conceptual overview of ATP6AP2/(P)RR in kidney development. Conditional loss-of-function studies establish developmental requirements for ATP6AP2 in specific renal lineages, including ureteric bud and stromal compartments [1,2,14,35]. ATP6AP2 association with V-ATPase and Wnt-related processes provides mechanistic context but does not demonstrate that prorenin/renin ligand-dependent signaling mediates the observed developmental phenotypes. Dot1/H3K79 findings should be interpreted within their tested experimental context. Connections to GDNF/RET, Notch, soluble (P)RR, and later-life renal disease are candidate or untested developmental links, not established direct regulatory effects. AI-assisted figure generation: ChatGPT-4 (OpenAI); scientific content and accuracy reviewed by the author.

Figure 2 summarizes developmental observations and candidate pathways. Solidly supported observations from ATP6AP2 loss-of-function studies must be distinguished from candidate downstream mechanisms; the figure is conceptual and does not establish direct regulation of every displayed pathway.

### 3.1. Kidney Development and Nephrogenesis

**Figure 3:**
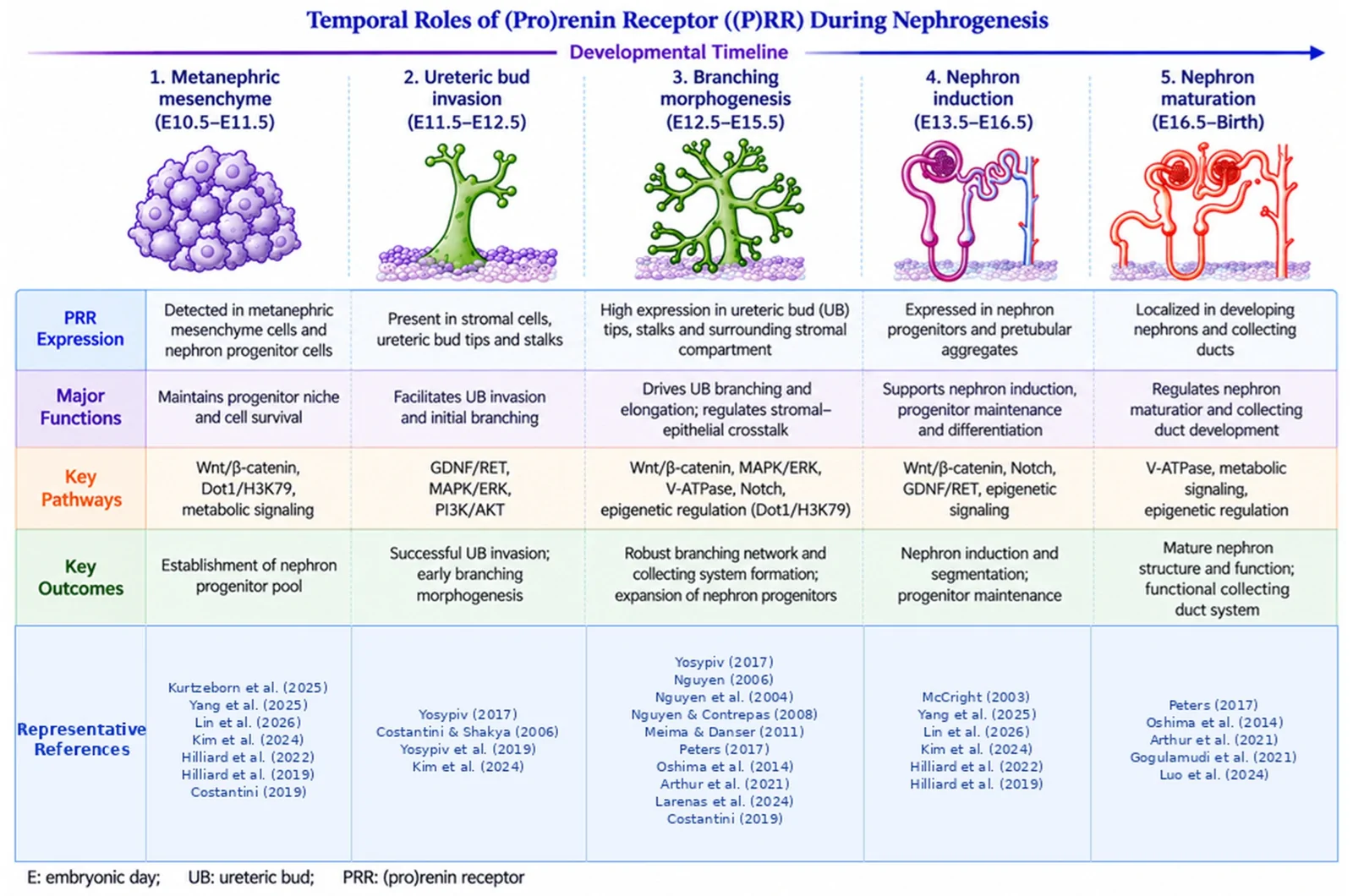
Temporal framework for ATP6AP2/(P)RR in nephrogenesis. The sequence of metanephric mesenchyme development, ureteric bud invasion and branching, nephron induction, and nephron maturation provides developmental context. Reported ATP6AP2 loss-of-function phenotypes should be attributed to the lineage and developmental stage actually tested. The placement of Wnt/β-catenin, V-ATPase, GDNF/RET, MAPK/ERK, Notch, or Dot1/H3K79 alongside a developmental stage does not itself establish direct, stage-specific regulation by ligand-bound (P)RR. AI-assisted figure generation: ChatGPT-4 (OpenAI); scientific content and accuracy reviewed by the author.

Temporal expression patterns and developmental functions of (P)RR during nephrogenesis are summarized in Figure 3. Kidney organogenesis requires tightly coordinated interactions between the ureteric bud and metanephric mesenchyme. Branching morphogenesis of the ureteric bud generates the collecting duct system, while nephron progenitors differentiate into mature nephron structures.

Previous studies demonstrated that kidney development involves reciprocal signaling between epithelial and mesenchymal tissues, while GDNF/RET signaling is essential for ureteric bud branching and nephron formation [3,4].

Lineage-targeted ATP6AP2 deletion studies reported abnormalities in renal morphogenesis and ureteric bud branching [1,2]. These findings demonstrate a requirement for ATP6AP2 in the tested models, but they do not establish whether the phenotype arises from prorenin/renin-dependent receptor activation, V-ATPase-associated homeostasis, or a combination of processes. The relevant developmental stage, targeted cell lineage, and specificity of the genetic manipulation must therefore be considered when comparing studies.

### 3.2. Ureteric Bud Branching Morphogenesis

Branching morphogenesis establishes the collecting duct tree and contributes to nephron endowment. Ureteric bud-targeted ATP6AP2 deletion has been associated with impaired branching and collecting duct abnormalities [1,2,35]. This is direct genetic evidence of a developmental requirement for ATP6AP2, not direct evidence that ligand-initiated (P)RR signaling regulates GDNF/RET or another specific branching pathway.

Song and Yosypiv reported that ureteric bud ATP6AP2 deletion was accompanied by inhibition of the Dot1/H3K79 pathway [35]. The finding supports a relationship between ATP6AP2 integrity and this epigenetic readout in the model, while the intermediate mechanism and contribution of V-ATPase-associated effects remain unresolved.

Together, these findings support a model in which (P)RR contributes to branching morphogenesis, epithelial differentiation, and developmental chromatin regulation [1,2,35]. The extent to which these effects reflect receptor-dependent signaling versus ATP6AP2/V-ATPase-associated cellular functions remains an important mechanistic question.

A separation-of-function approach is particularly important in ureteric bud epithelium, where secretory and endolysosomal trafficking support branching morphogenesis [11,12,13]. ATP6AP2 depletion may alter cell polarity, receptor localization, acidification, or viability upstream of many measured signaling readouts. An observed reduction in a pathway marker therefore cannot, on its own, distinguish a primary signaling defect from a secondary consequence of disrupted intracellular homeostasis. Rescue of branching with genetically defined ATP6AP2 variants and simultaneous measurements of V-ATPase function would help resolve this ambiguity.

Developmental timing adds a further layer of uncertainty in interpreting lineage-specific ATP6AP2 deletion findings [1,2,35]. A constitutive lineage-specific deletion can produce early defects that secondarily alter later cell composition, so a pathway change measured after tissue morphology has diverged may reflect a changed mixture of cell types rather than altered signaling within an otherwise equivalent population. Temporally inducible models and early time-course analyses would help identify the first detectable consequence of ATP6AP2 loss. Quantification of branching tips, epithelial proliferation, apoptosis, cell polarity, and organelle acidification in the same experimental series would provide a more informative phenotype than a single terminal histological comparison. When these assays are coupled with molecular readouts, it becomes possible to ask whether the pathway change precedes the morphological defect and whether restoring that pathway actually restores development. Such evidence is necessary before a mechanistic arrow is drawn as established in a summary figure.

### 3.3. Stromal (P)RR in Kidney Development

In addition to epithelial functions, stromal signaling plays a critical role during kidney organogenesis. Stromal cells regulate ureteric bud branching, nephron differentiation, and extracellular matrix organization [3,4].

Stromal ATP6AP2 deletion was reported to disrupt normal kidney development, with abnormalities affecting branching and nephron formation [14]. These lineage-targeted results support an important stromal requirement for ATP6AP2 but do not, by themselves, identify a ligand-dependent signaling cascade linking stromal ATP6AP2 to adjacent epithelium.

These findings suggest that stromal (P)RR contributes to epithelial–mesenchymal interactions necessary for proper renal development [14]. Disruption of stromal signaling may therefore contribute to developmental kidney abnormalities and reduced nephron endowment.

Stromal deletion studies are also informative because epithelial defects may be non-cell-autonomous [14]. Changes in extracellular matrix, secreted factors, stromal viability, or epithelial receptor distribution could each affect ureteric branching without direct binding between ATP6AP2 and a canonical branching ligand. Cell-specific transcriptomic profiling, tissue recombination, and rescue in the targeted stromal lineage would distinguish these alternatives more rigorously than bulk expression changes alone.

The epithelial and stromal compartments communicate bidirectionally during kidney development. Consequently, an altered epithelial branching pattern after stromal ATP6AP2 deletion may indicate disrupted production of stromal signals, changed matrix architecture, or altered physical support, rather than a receptor-mediated effect inside ureteric epithelial cells. Conversely, primary epithelial dysfunction can reshape the surrounding stroma. A mechanistic interpretation should therefore specify the manipulated lineage and the lineage in which each molecular or morphological endpoint was measured. Spatial transcriptomic or imaging approaches could distinguish a loss of a cell population from altered expression per cell. Tissue-specific rescue would be especially valuable, because it can test whether restoration in the targeted compartment is sufficient to normalize a phenotype. These experiments could also clarify whether apparently similar phenotypes in ureteric and stromal deletion models arise through shared mechanisms or through distinct routes that converge on branching morphogenesis.

### 3.4. Kidney Organoids and Emerging Developmental Models

Human-induced pluripotent stem cell-derived kidney organoids provide an experimental model for investigating ATP6AP2/(P)RR in human nephrogenesis. Lin et al. [21] demonstrated that transient (P)RR silencing during primitive streak formation produced smaller organoids, impaired proximal tubule development, increased stromal populations, and fibrotic changes, whereas podocyte and distal tubule development were largely unaffected. Silencing during nephron progenitor induction selectively impaired proximal tubule formation without reducing organoid size. These findings provide published experimental evidence for a stage-dependent requirement for (P)RR in human proximal tubule development, although they do not establish whether the effects are mediated by renin/prorenin binding, V-ATPase-associated functions, or other mechanisms.

Single-cell sequencing approaches have further improved understanding of developmental kidney cell populations. Comparative single-cell analyses identified both shared and divergent features between human and mouse kidney development [22]. Additional studies characterized chromatin and gene-regulatory dynamics of nephron progenitors during development [23,24].

These emerging technologies provide valuable tools for defining cell-type-specific functions of (P)RR and separating direct developmental effects from broader consequences of ATP6AP2/V-ATPase perturbation. Human organoid and single-cell models may also help assess the translational relevance of findings derived from animal systems.

Evidence from emerging model systems should be weighted by study design. An organoid perturbation may support human-cell relevance but may not reproduce vascularization, stromal diversity, maturation, or urinary flow found in vivo. Conversely, conditional mouse models provide intact tissue context but may exhibit compensation or unintended effects associated with the timing and specificity of Cre activity. A consistent developmental mechanism should ideally be reproduced across orthogonal models and supported by pathway-specific intervention rather than inference from similar morphology.

Human kidney organoids offer an opportunity to test whether ATP6AP2-associated processes observed in mice are conserved across species, but the evidentiary standard should remain high. Expression of ATP6AP2 in an organoid cell population does not establish that the protein regulates that population’s differentiation. Similarly, reduced organoid size or altered marker expression after perturbation could reflect generalized viability, proliferation, or lysosomal dysfunction. Experiments should include appropriate isogenic controls, independent perturbation methods, measurements of V-ATPase-related homeostasis, and rescue where feasible. The published organoid study [21] strengthens evidence for a human developmental role of (P)RR, particularly in proximal tubule formation, but its knockdown design does not distinguish ligand-dependent signaling from ATP6AP2/V-ATPase-associated cellular functions. The same caution applies to extrapolating from general single-cell kidney atlases, which define cellular states but do not by themselves demonstrate ATP6AP2-dependent causality.

Genetic mosaic approaches and developmental imaging provide complementary methods for examining renal morphogenesis and lineage-specific effects [37,38].

## 4. Developmental Signaling Pathways Associated with (P)RR

Table 1 distinguishes direct experimental findings, cellular associations, and proposed developmental pathways. A phenotype following ATP6AP2 deletion is direct evidence of a gene requirement, but it is not necessarily direct mechanistic evidence for ligand-dependent receptor signaling.

### 4.1. Wnt/β-Catenin Signaling

Wnt/β-catenin signaling is essential for nephron induction, nephron progenitor maintenance, and ureteric bud morphogenesis. Lineage-specific ATP6AP2 deletion studies provide experimental evidence that (P)RR loss is accompanied by altered Wnt/β-catenin signaling during renal development. In Six2-positive nephron progenitors, (P)RR deletion impaired progenitor proliferation and the inductive response to canonical Wnt/β-catenin signaling, resulting in reduced nephron formation and renal hypodysplasia [15]. In contrast, deletion of (P)RR in ureteric bud epithelial cells altered Wnt9b and Wnt11 expression and increased β-catenin transcriptional activity, alongside defective branching and collecting duct development [16].

These distinct lineage-dependent findings support an association between ATP6AP2 function and Wnt/β-catenin signaling during kidney development but do not establish a single shared mechanism. ATP6AP2/V-ATPase is also associated with Wnt signal-transduction machinery [11,12,13]. Further experiments are needed to distinguish primary changes in Wnt/β-catenin signaling from consequences of altered V-ATPase-dependent cellular homeostasis. Importantly, these phenotypes cannot be attributed specifically to renin/prorenin binding without selective mechanistic evidence.

### 4.2. Notch Signaling

Notch signaling plays a critical role in nephron patterning, segmentation, collecting duct differentiation, and progenitor cell-fate decisions during kidney development [5].

Notch is important in nephron patterning and differentiation [5], but the cited kidney-development literature does not demonstrate direct ATP6AP2-dependent regulation of Notch in this setting. Any Notch connection is therefore a testable hypothesis, not an established mechanism of (P)RR action.

### 4.3. GDNF/RET Signaling

GDNF/RET signaling is critical for ureteric bud branching and nephron induction. Costantini and Shakya demonstrated that disruption of RET signaling results in severe developmental kidney abnormalities.

GDNF/RET signaling is required for ureteric bud branching [4]. Because ATP6AP2 deletion also impairs branching in some models [1,2], GDNF/RET is a biologically plausible pathway to examine. Shared developmental phenotypes, however, do not demonstrate that ATP6AP2 enhances RET activity; ligand-specific epistasis and pathway-rescue experiments are needed.

### 4.4. Epigenetic Regulation and Chromatin Remodeling

Epigenetic regulation has emerged as an important mechanism controlling nephrogenesis and renal differentiation. Recent studies highlighted the importance of chromatin remodeling, histone modification, and developmental gene regulation during kidney development [25,26,34].

Nephron progenitor differentiation is regulated by dynamic chromatin remodeling and epigenetic signaling pathways [23,24,25,26,34]. Hilliard et al. characterized chromatin accessibility and gene-regulatory dynamics in nephron progenitors during kidney development.

In ureteric bud-targeted ATP6AP2 deletion models, altered Dot1/H3K79-associated signaling has been reported [35]. This provides a model-specific association between ATP6AP2 loss and an epigenetic pathway, but does not establish direct biochemical regulation of Dot1 by (P)RR or exclude an indirect effect of altered intracellular homeostasis.

These findings support a role for ureteric bud (P)RR in developmental epigenetic regulation through the Dot1/H3K79 pathway. Further work is needed to determine the cell-type specificity, temporal requirements, and relationship of this effect to ATP6AP2/V-ATPase function.

The Dot1/H3K79 finding illustrates why evidence categories matter: a change in histone-associated pathway measurements after ATP6AP2 deletion is stronger than a purely speculative association, but still does not prove direct molecular interaction between ATP6AP2 and a chromatin-modifying enzyme. Distinguishing primary from secondary effects would require temporally resolved epigenetic profiling, analysis of Dot1 abundance and activity, and rescue of the chromatin phenotype without broadly restoring unrelated cellular defects.

Chromatin changes can arise downstream of altered metabolism, differentiation state, or stress, making temporal order particularly important when evaluating ATP6AP2-associated epigenetic observations. For example, a decrease in a histone modification following deletion may be compatible with altered methyltransferase activity, but it could also reflect altered abundance of cells carrying that mark or broader disturbances in chromatin regulation. Cell-resolved profiling and assays of enzyme abundance and activity would help discriminate among these possibilities. Furthermore, a developmental rescue that restores branching while leaving the histone readout abnormal, or restores the histone readout without rescuing branching, would challenge a simple linear mechanism. Until such experiments are available, the appropriate claim is that ureteric bud ATP6AP2 deletion is associated with altered Dot1/H3K79-related signaling in the reported model [35]. This is a meaningful finding without being proof of a direct receptor-to-chromatin signaling pathway.

### 4.5. Soluble (P)RR in Kidney Development and Disease

Soluble (P)RR has increasingly been implicated in renal physiology and disease progression. Recent studies emphasized the role of soluble (P)RR in intrarenal RAS activation, sodium handling, fibrosis, and hypertension [17,18,19,20].

Experimental studies demonstrated that collecting duct-derived (P)RR contributes to renovascular hypertension, ischemic nephropathy, and activation of intrarenal RAS signaling during kidney injury [40,41].

High-glucose conditions induce (P)RR trafficking and stimulate profibrotic signaling in collecting duct cells, including pathways involving TGF-β and CTGF [32,33].

Evidence for s(P)RR in renal sodium transport, hypertension, and injury is primarily derived from physiological or disease models [17,18,19,20,40,41]. No cited study establishes that s(P)RR directly regulates nephron formation. Developmental functions and a causal developmental-to-adult disease connection should therefore be presented as questions for future testing.

Nephron (P)RR deficiency has also been associated with changes in renal medullary endothelin signaling, an adult physiological observation that does not by itself establish a developmental mechanism [42]. Circulating s(P)RR has been examined in relation to kidney function in young adults, but such associations do not establish causality in nephrogenesis [43].

## 5. (P)RR in Kidney Disease and Developmental Programming

Increasing evidence supports a role for (P)RR in multiple kidney diseases, including diabetic nephropathy, fibrosis, inflammation, ischemic injury, and hypertension.

Experimental studies demonstrated that (P)RR contributes to renal mitochondrial dysfunction, apoptosis, and fibrosis in diabetic kidney disease [28]. Additional studies reported that (P)RR mediates tubular epithelial pyroptosis through DPP4-JNK signaling pathways during diabetic kidney disease [31].

(P)RR also mediates inflammatory responses during renal ischemia and contributes to cyclosporin A-induced nephropathy [29,30].

A reduced nephron complement can increase vulnerability to hypertension or chronic kidney disease in general developmental-programming frameworks [2,6]. Whether a developmental ATP6AP2 perturbation itself causes these adult outcomes remains unknown. Adult renal injury studies [28,29,30,31,32,33,40,41] and developmental knockout studies [1,2,14,35] involve distinct interventions and time frames; they should not be combined into a demonstrated longitudinal causal chain.

Reviews have examined (P)RR in hypertension and metabolic regulation [44], while experimental therapeutic studies have evaluated receptor-targeted interventions in kidney disease [45,46]. These adult disease findings should not be interpreted as evidence of developmental ATP6AP2-specific mechanisms.

## 6. Integrated Developmental and Disease Model of (P)RR

**Figure 4:**
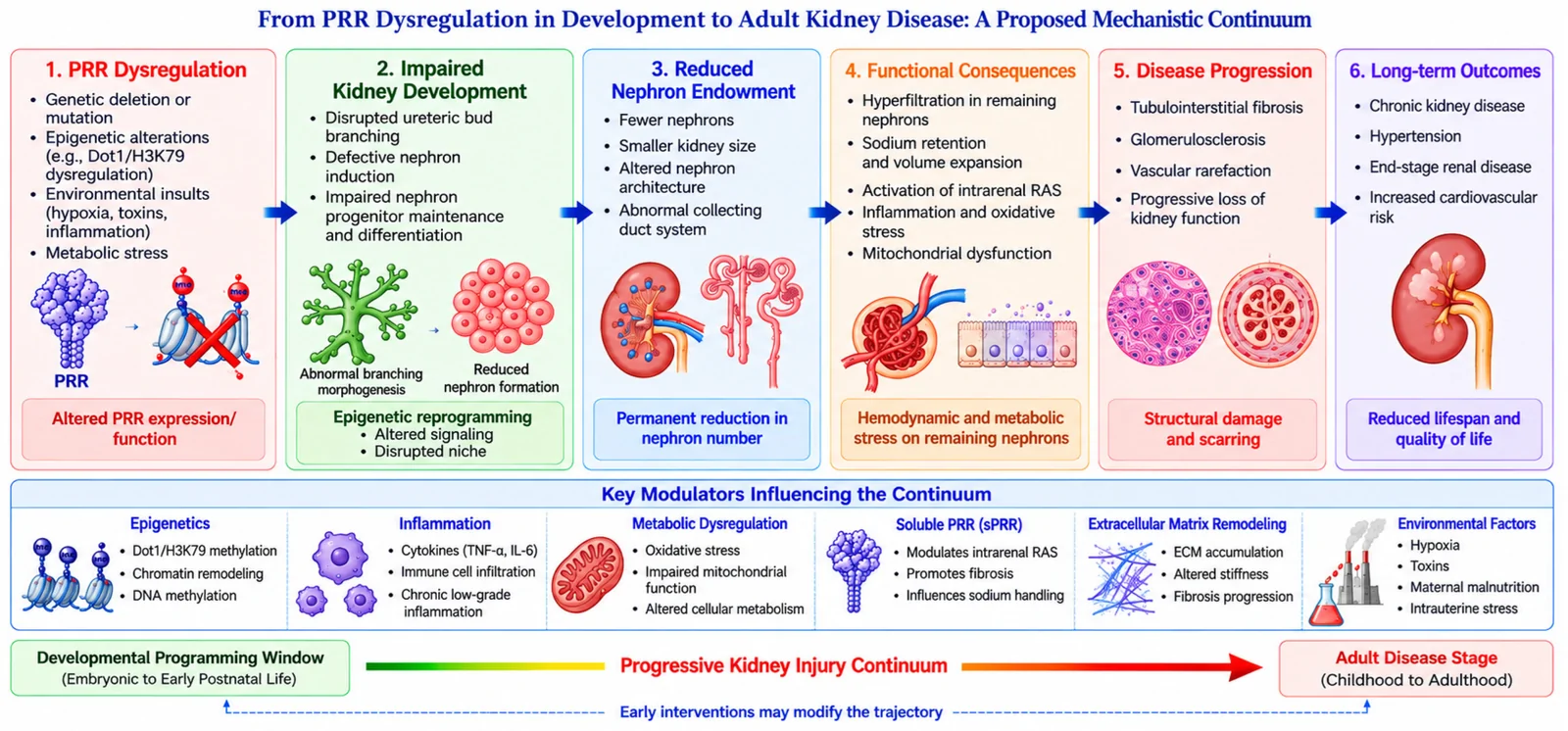
Hypothesis-generating framework relating developmental ATP6AP2 perturbation to potential later renal vulnerability. Kidney developmental phenotypes after ATP6AP2 perturbation and observations concerning adult renal injury or disease derive from different experimental contexts. A progression from impaired development to reduced nephron endowment, hyperfiltration, inflammation, fibrosis, chronic kidney disease, or end-stage renal disease has not been demonstrated longitudinally as a consequence of developmental ATP6AP2 deletion. Any connecting arrows represent hypotheses requiring direct testing, not established causal transitions. AI-assisted figure generation: ChatGPT-4 (OpenAI); scientific content and accuracy reviewed by the author.

The integrated model should be read as two evidence domains: developmental ATP6AP2 perturbations are associated with renal morphogenetic defects, whereas adult kidney disease studies implicate ATP6AP2/(P)RR or s(P)RR in injury, sodium transport, and fibrosis. A direct causal bridge from the former to the latter has not been established. Figure 4 is therefore a hypothesis-generating comparison, not a demonstrated temporal progression.

Developmental ATP6AP2 perturbation has been associated with branching and differentiation defects in targeted models [1,2,14,35]. Separately, adult experimental studies implicate (P)RR-related processes in sodium retention, inflammation, and fibrotic responses [17,18,19,20,28,29,30,31,32,33,40,41]. Whether early ATP6AP2 disruption leads to later hyperfiltration, hypertension, CKD, or ESRD has not been demonstrated by the cited longitudinal evidence. The illustration therefore represents a research hypothesis rather than a causal continuum.

In established renal physiology and disease models, s(P)RR has been associated with intrarenal RAS activation, sodium handling, fibrosis, inflammation, and metabolic dysregulation. Whether these actions mechanistically connect developmental abnormalities to progressive adult renal disease remains an open question. The major developmental and pathological functions of (P)RR in kidney structures and developmental processes are summarized in Table 2.

Interpretive boundaries: First, an embryonic branching phenotype does not quantify adult nephron number unless nephron endowment is directly measured. Second, adult hypertension, hyperfiltration, and fibrosis can arise through many routes unrelated to developmental ATP6AP2 perturbation. Third, injury-associated s(P)RR measurements cannot retrospectively establish a causal embryonic exposure. A convincing developmental-programming claim requires temporal control of the intervention, appropriately matched controls, direct nephron quantification, and follow-up of blood pressure and kidney function after maturation. Such studies are currently a research priority rather than an established conclusion.

A second distinction concerns the use of adult disease literature to motivate developmental hypotheses. Studies of diabetic injury, renal ischemia, sodium retention, and fibrosis identify functions of ATP6AP2-related pathways in mature kidneys under specific stresses [17,18,19,20,28,29,30,31,32,33,40,41]. They cannot establish that a transient developmental alteration of ATP6AP2 causes the same processes decades or months later. Reduced nephron endowment is a biologically credible risk factor in broader renal developmental research, but its presence and magnitude must be demonstrated in the particular ATP6AP2 model. Moreover, kidney injury in adulthood may depend on systemic blood pressure, compensatory growth, sex, age, and environmental exposures. Longitudinal developmental models should therefore distinguish a persistent structural deficit from a later injury response and should evaluate both renal function and pathology over time. Figure 4 should depict the developmental and adult evidence as parallel domains connected by an explicitly untested hypothesis, rather than by an uninterrupted causal sequence.

The experimental evidence from developmental ATP6AP2 loss-of-function models and distinct adult renal physiology models is compared in Table 3, highlighting the conclusions supported by each model and their mechanistic limitations.

## 7. Emerging Technologies and Future Directions

Kidney organoids, lineage-restricted gene manipulation, single-cell and spatial transcriptomics, and chromatin profiling can define the timing and cell specificity of ATP6AP2 requirements. Crucially, these approaches should be combined with functional assays of lysosomal acidification, endosomal trafficking, and V-ATPase assembly to distinguish broad cellular dysfunction from signaling-specific phenotypes. Developmental changes in gene expression alone do not demonstrate direct pathway activation or repression.

Studies of (P)RR and s(P)RR in pregnancy and maternal renin–angiotensin regulation provide a separate physiological context [47,48]; any connection to fetal kidney ATP6AP2-dependent morphogenesis remains to be tested.

A decisive experimental strategy would compare wild-type ATP6AP2 rescue with separation-of-function variants that preserve V-ATPase association but disrupt ligand binding, or vice versa, where technically validated. Additional tests could include prorenin/renin ligand perturbation, receptor-selective inhibitors with appropriate specificity controls, lineage-specific epistasis, and ex vivo ureteric bud branching assays. For GDNF/RET and Notch, direct pathway readouts and rescue experiments are needed. To evaluate developmental programming, longitudinal cohorts must follow renal structure, nephron endowment, blood pressure, and renal function into adulthood, rather than infer adult disease from embryonic phenotypes.

Therapeutic targeting of (P)RR also requires caution. Selective blockers or decoy approaches may offer experimental or future therapeutic opportunities in renal disease, but ATP6AP2 has essential cellular functions, including its association with V-ATPase. Any translational strategy will therefore require careful evaluation of target specificity, developmental timing, cell-type selectivity, efficacy, and safety.

A practical evidence hierarchy for future studies would be: (1) reproducible developmental phenotype following cell-specific ATP6AP2 perturbation; (2) altered pathway activity in the relevant cells at a time preceding tissue disruption; (3) pathway-specific genetic or pharmacological rescue; and (4) separation-of-function experiments showing whether ligand binding or V-ATPase-related functions are necessary. This hierarchy also prevents conflation of a disease-model association with a developmental mechanism. Negative findings and non-rescued phenotypes should be reported, because they are essential to excluding attractive but incorrect causal models.

## 8. Conclusions

The evidence supports ATP6AP2 as an important contributor to renal development, especially in lineage-specific genetic models of ureteric bud and stromal function [1,2,14,35]. The most defensible conclusion is a developmental requirement for ATP6AP2, not a uniform ligand-dependent mechanism across all proposed signaling pathways. Wnt/V-ATPase interactions and Dot1/H3K79-associated changes warrant mechanistic study; direct regulation of GDNF/RET, Notch, and several other proposed pathways remains unproven.

Membrane-associated ATP6AP2, ligand-dependent (P)RR signaling, V-ATPase-related cellular functions, and soluble (P)RR must be considered separately. Likewise, observations from adult injury and sodium-handling models cannot establish a developmental cause of adult renal disease.

Kidney organoids, single-cell and epigenetic approaches now provide the tools needed to resolve these uncertainties. Future studies that define cell-specific mechanisms, separate receptor signaling from V-ATPase-associated functions, and test developmental-to-adult outcomes will be essential for determining whether (P)RR pathways can be translated safely into strategies for congenital or acquired kidney disease.

## Figures and Tables

**Table 1:** Evidence-graded pathways and cellular processes associated with ATP6AP2/(P)RR. “Direct” refers to a tested gene requirement or biochemical/cellular relationship in the indicated context; it does not imply that prorenin binding is the cause of an ATP6AP2-deletion phenotype.

Pathway	Role of (P)RR	Developmental/Cellular Function	Kidney Structures Affected	Representative References	Evidence Level/Limitation
Wnt/β-catenin	ATP6AP2/V-ATPase is associated with Wnt signal transduction; kidney-specific mediation requires testing.	Cell fate and nephron induction	Nephron progenitors, epithelium	[11,12,13,15,16,23,24,25,26]	Lineage-specific Wnt/β-catenin changes demonstrated after ATP6AP2 deletion; primary mechanism unresolved
GDNF/RET	No direct enhancement of RET by (P)RR established in cited renal developmental studies.	Ureteric bud branching	Ureteric bud	[3,4,14]	Proposed; shared phenotype is insufficient
MAPK/ERK	Prorenin/(P)RR-associated MAPK responses reported outside defined developmental models.	Proliferation and stress responses	Context dependent	[7,8,9,10]	Ligand-linked cellular evidence; renal developmental link indirect
Notch	No direct kidney developmental regulation by ATP6AP2 established.	Nephron patterning	Developing nephrons	[5]	Proposed pathway
V-ATPase function	ATP6AP2-associated acidification and trafficking functions.	Organelle homeostasis	Renal epithelia	[11,12,13,39]	Established cellular association; mechanism of knockout phenotype unresolved
Dot1/H3K79	Altered pathway readout after ureteric bud ATP6AP2 deletion.	Chromatin-associated developmental regulation	Ureteric bud	[35]	Direct deletion-model association; biochemical mechanism unknown
PI3K/AKT	Pathway implicated in (P)RR-related signaling outside a defined renal developmental mechanism.	Cell survival	Context dependent	[7,8,9,10,11,12,13]	Indirect; developmental role not demonstrated
TGF-β/CTGF	High-glucose collecting duct models report profibrotic changes.	Adult injury/fibrosis	Collecting duct cells	[32,33]	Disease-model association; not developmental
DPP4–JNK	Reported in diabetic tubular injury models.	Pyroptosis	Tubular epithelium	[31]	Disease-model mechanism; not developmental
Intrarenal RAS	s(P)RR/(P)RR implicated in sodium transport and RAS regulation.	Sodium balance	Collecting duct	[17,18,19,20,40,41]	Physiological/disease evidence; developmental link unknown
Metabolic signaling	Metabolic control of renal progenitors is known; specific ATP6AP2 regulatory axis not established.	Progenitor metabolism	Nephron progenitors	[6,23,24,25,26]	Proposed ATP6AP2 link
Inflammatory signaling	ATP6AP2-associated injury responses in adult models.	Inflammation	Tubular epithelium	[28,29,30]	Disease-model association; not developmental

**Table 2:** Evidence-aware summary of developmental ATP6AP2 requirements, proposed signaling mechanisms, and distinct adult renal disease observations.

Kidney Structure/Developmental Process	Role of (P)RR	Major Pathways/Mechanisms	Developmental or Pathological Outcome	Key References
Ureteric bud branching morphogenesis	Promotes ureteric bud branching and elongation	GDNF/RET, Wnt/β-catenin, MAPK/ERK	Proper collecting duct formation and nephron endowment	[1,2,4,35]
Collecting duct development	Regulates epithelial differentiation and tubular maturation	V-ATPase signaling, intracellular trafficking	Normal collecting duct maturation and tubular organization	[1,12,13,40,41]
Stromal cell signaling	Maintains stromal–epithelial communication during nephrogenesis	Growth factor regulation, extracellular signaling	Supports nephron induction and branching morphogenesis	[14]
Nephron progenitor maintenance	Supports progenitor survival, self-renewal, and differentiation	Wnt/β-catenin, Notch, metabolic signaling	Maintains nephron progenitor pool and nephron formation	[6,15,22,23,24,25,26]
Nephron differentiation and maturation	Regulates nephron segmentation and epithelial maturation	Developmental transcriptional regulation	Proper nephron structure and function	[1,2,5,25,26]
Epigenetic regulation during kidney development	Modulates chromatin remodeling and developmental gene expression	Dot1/H3K79 methylation, chromatin accessibility	Controls nephrogenesis and developmental programming	[23,24,25,26,34,35,36]
V-ATPase-associated cellular regulation	Maintains intracellular acidification and vesicle trafficking	V-ATPase complex signaling	Cellular homeostasis and developmental regulation	[11,12,13,39]
Soluble (P)RR generation	Produced through proteolytic cleavage of membrane PRR	Protease-mediated ectodomain shedding	Release of biologically active soluble PRR	[17,18,19,20]
Renal sodium handling	Regulates sodium reabsorption in collecting ducts	Intrarenal RAS activation	Blood pressure and fluid balance regulation	[19,20,40]
Renal fibrosis	Promotes profibrotic signaling under stress conditions	TGF-β, CTGF, MAPK/ERK pathways	Fibrosis and chronic kidney disease progression	[28,32,33]
Diabetic kidney disease	Mediates mitochondrial dysfunction and tubular injury	DPP4-JNK, oxidative stress, apoptosis	Diabetic nephropathy progression	[28,31]
Renal ischemia and inflammation	Contributes to inflammatory signaling and injury response	Inflammatory cytokine pathways	Ischemic kidney injury and renal inflammation	[29,30]
Kidney organoid development	Transient (P)RR silencing disrupts proximal tubule development in human kidney organoids	Stage-dependent proximal tubule differentiation; mechanism unresolved	Published human organoid developmental phenotype	[21]
Developmental programming of kidney disease	Hypothesized connection; not tested longitudinally	Nephron endowment as a general risk framework	Adult hypertension/CKD after developmental ATP6AP2 deletion not demonstrated	[2,6,17,18,19,20]

**Table 3:** Comparison of developmental ATP6AP2 loss-of-function models and distinct adult physiology models. This is a qualitative summary; primary studies should be consulted for precise genotype, Cre driver, developmental timing, and sample sizes.

Model/Intervention	Reported Phenotype or Observation	What the Evidence Supports	References
Ureteric bud-targeted ATP6AP2 deletion in mice	Abnormal branching/collecting duct development	Direct genetic requirement in targeted lineage; ligand dependence untested	[1,2,35]
Stromal ATP6AP2 deletion in mice	Renal developmental abnormalities and disrupted stromal–epithelial interactions	Direct stromal genetic requirement; downstream signaling not resolved	[14]
Ureteric bud ATP6AP2 deletion; Dot1/H3K79 readout	Reduced Dot1/H3K79-associated signaling	Association in knockout context, not direct enzyme regulation	[35]
Transient (P)RR silencing in human kidney organoids	Impaired proximal tubule development; timing-dependent organoid effects	Published human organoid evidence; ligand dependence versus V-ATPase function unresolved	[21]
Collecting duct s(P)RR/adult physiology models	Sodium transport and cardiorenal phenotypes	Adult physiology, not a nephrogenesis model	[17,18,19,20,34,40,41]

## Data Availability

No new data were created or analyzed in this study. Data sharing is not applicable to this article.
